# Indocyanine Green Fluorescence Imaging for Evaluation of Uterine Blood Flow in Cynomolgus Macaque

**DOI:** 10.1371/journal.pone.0035124

**Published:** 2012-04-20

**Authors:** Iori Kisu, Kouji Banno, Makoto Mihara, Li-Yu Lin, Kosuke Tsuji, Megumi Yanokura, Hisako Hara, Jun Araki, Takuya Iida, Takayuki Abe, Keisuke Kouyama, Nobuhiko Suganuma, Daisuke Aoki

**Affiliations:** 1 Department of Obstetrics and Gynecology, School of Medicine, Keio University, Tokyo, Japan; 2 Department of Plastic and Reconstructive Surgery, Graduates School of Medicine, University of Tokyo, Tokyo, Japan; 3 The Center for Clinical Research, School of Medicine, Keio University, Tokyo, Japan; 4 Department of Human Health Sciences, Kyoto University Graduate School of Medicine, Kyoto, Japan; Konkuk University, Republic of Korea

## Abstract

**Background:**

Uterine blood flow is an important factor in uterine viability, but the number of blood vessels required to maintain viability is uncertain. In this study, indocyanine green (ICG) fluorescence imaging was used to examine uterine hemodynamics and vessels associated with uterine blood flow in cynomolgus macaque.

**Methods:**

The uterus of a female cynomolgus macaque was cut from the vaginal canal to mimic a situation during trachelectomy or uterine transplantation surgery in which uterine perfusion is maintained only with uterine and ovarian vessels. Intraoperative uterine hemodynamics was observed using ICG fluorescence imaging under conditions in which various nutrient vessels were selected by clamping of blood vessels. A time-intensity curve was plotted using imaging analysis software to measure the T_max_ of uterine perfusion for selected blood vessel patterns. Open surgery was performed with the uterus receiving nutritional support only from uterine vessels on one side. The size of the uterus after surgery was monitored using transabdominal ultrasonography.

**Results:**

The resulting time-intensity curves displayed the average intensity in the regions of the uterine corpus and uterine cervix, and in the entire uterus. Analyses of the uterine hemodynamics in the cynomolgus macaque showed that uterine vessels were significantly related to uterine perfusion (P = 0.008), whereas ovarian vessels did not have a significant relationship (P = 0.588). When uterine vessels were clamped, ovarian vessels prolonged the time needed to reach perfusion maximum. Postoperative transabdominal ultrasonography showed that the size of the uterus was not changed 2 months after surgery, with recovery of periodic menstruation. The cynomolgus macaque has got pregnant with favorable fetus well-being.

**Conclusion:**

Uterine vessels may be responsible for uterine blood flow, and even one uterine vessel may be sufficient to maintain uterine viability in cynomolgus macaque. Our results show that ICG fluorescence imaging is useful for evaluation of uterine blood flow since this method allows real-time observation of uterine hemodynamics.

## Introduction

The uterine artery and the ovarian/vaginal arteries, which connect with branches of the uterine artery, contribute to blood supply to the uterus, and many studies on the dominant regions of the arteries have been performed [1]–[4]. Radical trachelectomy is performed for young women with early cervical cancer for preservation of fertility [5] and uterine blood flow is an important factor in maintenance of uterine function in this procedure. Several studies have suggested that preservation of the uterine artery is required to maintain the potential for pregnancy and child birth [6]–[9]. Insufficient uterine blood flow might cause premature birth, intrauterine growth retardation, or increased susceptibility to infection [10], [11], but a clear conclusion has not been reached.

Human uterine transplantation was first performed in 2000 [12]. This transplantation may permit pregnancy and child birth for women with congenital infertility, such as that produced by defects of the uterus and vagina in Mayer-Rokitansky-Küster-Hauser syndrome [13], and for those with acquired uterine infertility caused by hysterectomy due to treatment for a malignant uterine tumor [14], [15], benign diseases [16] such as uterine myoma and uterine adnomyosis, and severe postpartum bleeding [17]. Uterine transplantation has also been studied in mouse [18]–[20], rat [21]–[23], pig [24], [25], sheep [26], [27], cynomolgus macaque [28], [29], and baboon [30], but pregnancy in non-human primates after transplantation has not been achieved, including after autotransplantation. This may be due to the complicated surgical techniques, ischemic reperfusion injury, and the need for ischemic preservation. Selection of the type and number of blood vessels required to maintain uterine blood flow may also be important. However, it is unclear how many arteries and veins are required to maintain perfusion of a transplanted uterus.

X-ray angiography and lymphoscintigraphy are used for conventional non-invasive diagnostic imaging of organs and blood/lymph vessels in vivo, but radiation exposure and the large size and cost of the equipment are problematic. To solve these problems, an indocyanine green (ICG) fluorescence imaging system was developed and used by Kitai et al. for sentinel lymph node mapping in patients with breast cancer [31]. Since lymph and blood flow can be observed in real time during surgery using ICG fluorescence imaging, the use of this approach has expanded to sentinel lymph node mapping in patients with breast cancer [32] and digestive organ cancer [34], [35], evaluation of lymph channels in patients with lymphedema [35], evaluation of graft vessels [36] in coronary bypass surgery, and blood flow evaluation in reconstructed organs such as free flaps [37], [38]. Thus, this angiographic technique is used widely. However, in obstetrics and gynecology, it has only been used for observation of placental communicating vessels in patients with twin-twin transfusion syndrome [39] and for observation of an angiostomy site and uterine blood flow in our experiments on uterine transplantation research [29].

In this study, we used ICG fluorescence imaging for evaluation of uterine blood flow in cynomolgus macaque to examine uterine hemodynamics, with the goal of understanding the important factors in maintenance of uterine viability in radical trachelectomy and uterine transplantation.

## Methods

### Animals

The study was performed in a 7-year-old female cynomolgus macaque (weight: 3.4 kg) with a regular menstrual cycle and a history of child birth. The cynomolgus macaque is anatomically and physiologically close to humans. The body and tail lengths are 38–55 and 40–65 cm, respectively, in adults, and the body weight is 5–9 kg in males and 3–6 kg in females, which are about one-tenth of those of humans. The cynomolgus macaque has a menstrual cycle of approx. 1 month and is a continuous breeder throughout the year, with a gestational period of approx. 165 days. The anatomical characteristics of the internal genital organs, including the uterus and adnexa, and vascular system of female cynomolgus macaques are similar to those of humans [28]. This study was carried out in strict accordance with the recommendations in the Guide for the Care and Use of Laboratory Animals of the National Research Council. The study protocol was approved by the Institutional Scientific Evaluation and Review Committee and the Animal Care and Use Committee of the Institute of Primate Research, Shin-Nihon-Kagaku, Kagoshima, Japan (Permit Number: SBL740-001), which is fully accredited by Association for Assessment and Accreditation of Laboratory Animal Care International (Approval No. 001404). The cynomolgus macaque was reared in each cage under the following environmental conditions: temperature of 23–29°C, humidity of 35–75%, lighting of 12 hr/day, and toys to play with all day long. The animal was also fed a commercial monkey diet once daily, with supplemental fruits and vegetables seven times weekly both pre and post operatively. Lactated Ringer’s solution was administered i.v. if there was a change in the post-operative condition, such as appetite loss or dehydration.

### Anesthesia

After sedation with atropine sulfate (0.02 ml/kg, i.m. injection; Mitsubishi Tanabe Pharma Corp., Tokyo, Japan) and ketamine hydrochloride (50 mg/ml aqueous solution, 0.2 ml/kg i.m.; Kamud Drugs, Tokyo, Japan), the abdomen was shaved and the animal was maintained in the supine position. The abdomen was disinfected with 70% ethanol and iodine tincture and covered with a sterilized drape. A tracheal tube was inserted and anesthesia was maintained with isoflurane inhalation (0.5–1.0%; Abbott Japan, Tokyo, Japan). During surgery, anesthesia was maintained with inhalation of a mixture of carrier gas (nitrous oxide/oxygen, 2∶1) and 1% isoflurane using an inhalant anesthesia vaporizer for isoflurane (AIV-5; Kimura Medical Co., Tokyo, Japan) and equipment for general anesthesia (Kimura General Anesthesia Device Compact-15, Kimura Medical Co.) under spontaneous respiration. To compensate for fluid loss during surgery, the animal received continuous i.v. infusion of 0.163 M NaCl at 3–4 ml/kg/h. Antibiotics (dihydrostreptomycin sulfate 0.02–0.1 ml/kg i.m.; Meiji Seika, Tokyo, Japan) and buprenorphine hydrochloride (Lepetan 0.005–0.001 mg/kg i.m.; Otsuka Pharmaceutical Co., Tokyo, Japan) were administered after surgery to prevent infection and to treat pain.

### Surgical Techniques

All surgical procedures were performed using sterile techniques and surgical loupes. An abdominal midline longitudinal incision (from the pubic bone up to the level of the umbilicus) was performed. The intestines were packed into the upper abdomen. After confirming that the anatomical characteristics of the internal genital organs and vascular system of the cynomolgus macaque were similar to those of humans, we cut the round ligament and examined the broad ligaments of the uterus and retroperitoneum. After identifying and exfoliating the uterine artery/vein, ovarian artery/vein, and urinary duct for visual confirmation, we then exfoliated the urinary bladder and rectum from the uterus. Assuming performance of radical trachelectomy or uterine transplantation, we treated the parametrium and paracorpium at the height of the vaginal side, rather than at the height of the uterine artery/vein because this would block blood flow from the vagina. With the uterine artery/vein and ovarian artery/vein connected to the uterus, the vaginal canal was cut. ICG (Diagnogreen 0.5%; Daiichi Pharmaceutical, Tokyo, Japan) was then intravenously administered and ICG fluorescence imaging was used to observe perfusion of the uterus in real time. After completion of observations, the base of the ovarian duct was cut so that both adnexa remained on the side of the ovarian artery/vein. Then, the uterine artery/vein on one side was cut so that only the uterine artery/vein on the other side could serve as nutrient vessels. The disconnected vaginal canal was anastomosed and suture of the retroperitoneum and repair of the round ligament were performed before closing the abdominal incision.

### Intraoperative ICG Fluorescent Angiography

**Figure 1:**
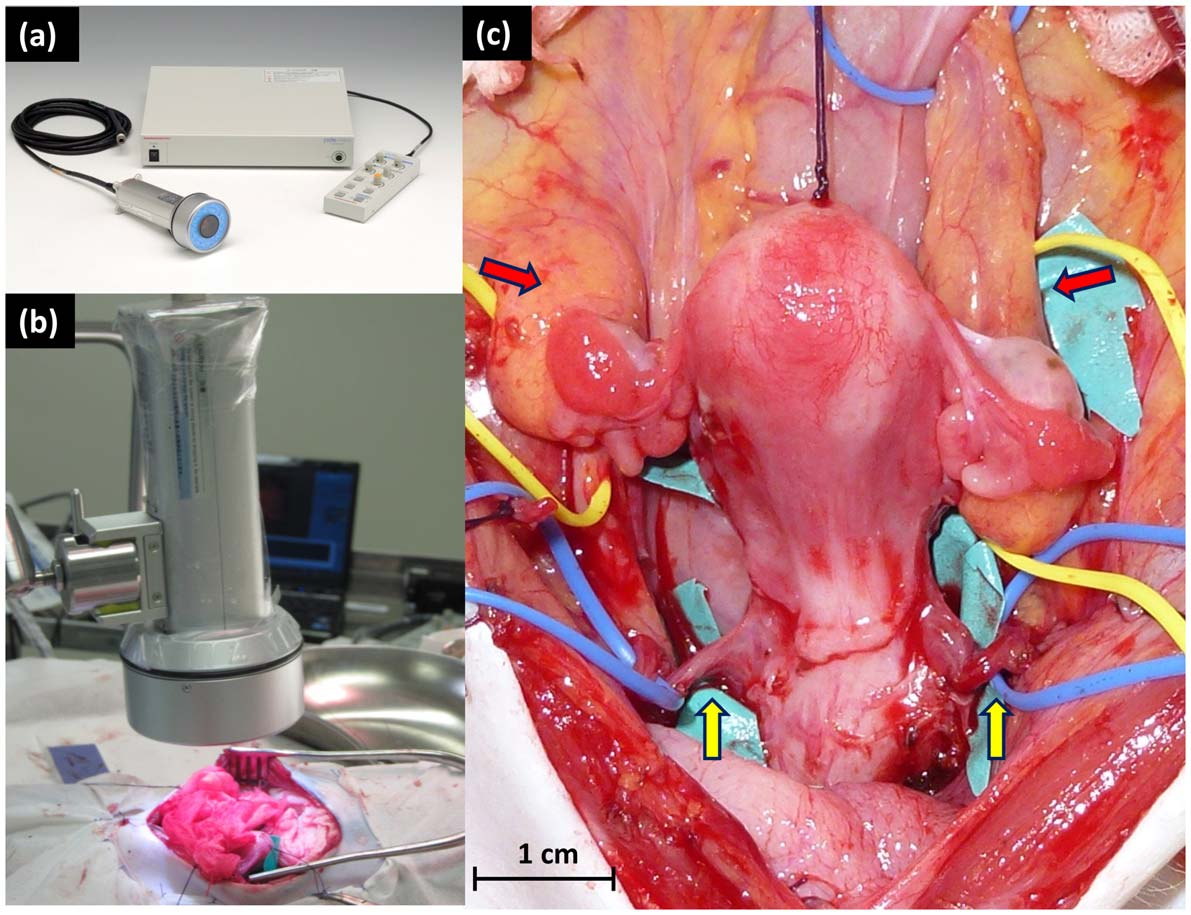
Preparation for intraoperative observation of uterine hemodynamics with ICG fluorescence imaging. (a) The ICG fluorescence imaging system, including the main body, camera unit, and remote controller. (b) CCD camera unit of a PDE installed at the site of surgery. (c) The uterus with a separated vaginal canal. The uterus was connected by the uterine artery/vein (yellow arrow) and ovarian artery/vein (red arrow).

ICG is a dark blue-green soluble compound with a molecular weight of 774.96. ICG binds with plasma protein and is distributed to blood vessels throughout the body, taken into the liver, and excreted in bile from the liver as a free compound. ICG absorbs near infrared (IR) rays of 750–800 nm and produces strong fluorescence of 840 nm when excited [40]. However, since this wavelength is within the range of near IR, fluorescence images cannot be observed with the naked eye. The Photodynamic Eye (hereinafter referred to as PDE; Hamamatsu Photonics KK, Hamamatsu, Japan) produces excitation light of 760 nm from a light emitting diode and enables visual imaging with a CCD through an optical filter, which blocks excitation light of ≤820 nm and transmits only fluorescence (Fig. 1a). Using this IR observation camera system, ICG can be observed during surgery, which allows monitoring of vascular and lymph dynamics under the tissue surface and observation of the distribution of the fluorescent reagent in tissues.

### Intraoperative Observation of Uterine Hemodynamics with ICG Fluorescence Imaging

ICG (0.25 mg/ml, 1 ml) was intravenously administered from the periphery. The CCD camera with a PDE was installed in the surgical field and real-time images of uterine perfusion during surgery were obtained by ICG fluorescence imaging and observed on the monitor of a laptop computer (Fig. 1b). Uterine hemodynamics was observed after clamping blood vessels to examine perfusion from selected vessels, as shown in Table 1. Uterine perfusion was examined for vessel patterns with 6 vessels (A), 4 vessels (B), 2 vessels, including uterine vessels only (C), ovarian vessels only (D), a pair of uterine and ovarian vessels on the same side (E) and the opposite side (F), one ovarian vessel (G), and one uterine vessel (H) (Table 1). Other than for pattern A, hemodynamics was observed under conditions in which the vaginal canal was completely separated from the uterus (Fig. 1c). All experiments were performed with systolic blood pressure 90–99 mmHg and diastolic blood pressure 40–49 mmHg to ensure that the ICG fluorescence distribution was not affected by blood pressure.

**Table 1:** Patterns of selected blood vessels used for uterine perfusion observed by ICG fluorescence imaging.

Pattern	Uterine vessels		Ovarian vessels		Vaginal vessels		Number of patent vessels	
	Rt.	Lt.	Rt.	Lt.	Rt.	Lt.	Uterine	Ovarian
A	+	+	+	+	+	+	2	2
B	+	+	+	+			2	2
C	+	+					2	0
D			+	+			0	2
E	+		+				1	1
F	+			+			1	1
G			+				0	1
H	+						1	0

+: Vessel patency.

### ROI Software

**Figure 2:**
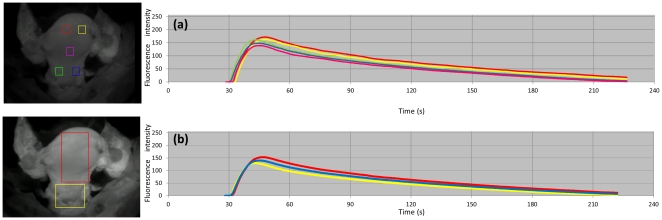
Time-intensity curves prepared by ROI software to show temporal changes in ICG fluorescence intensity (Pattern A). (a) Temporal changes in intensity in 5 specified regions (red: right uterine corpus, yellow: left uterine corpus, green: right uterine cervix, blue: left uterine cervix, pink: uterine isthmus) (b) Temporal changes in average intensity in the uterine corpus (red), uterine cervix (yellow) regions, and the whole uterine region including the corpus and cervix regions (blue).

Images were imported into a computer and changes in fluorescence intensity were measured over time using image intensity analysis software (ROI [Region Of Interest] software: Hamamatsu Photonics KK). This software analyzes the intensity of images on a scale of 0 to 256. Changes in average intensity over time in a given region are shown numerically and can be plotted graphically using the software (Fig. 2a, Fig. S1). In ICG images for the patterns of blood vessels shown in Table 1, individual regions were defined using the ROI software to include the uterine corpus and cervix. These data and the average intensity in the entire uterine region, including both corpus and cervix regions, were plotted as time-intensity curves (Fig. 2b). The time (s) at which the intensity reached a maximum (T_max_) was measured as a marker of the blood supply to the uterus. The half-life of the highest intensity (T_1/2_) was also measured for evaluation of uterine blood perfusion.

### Postoperative Observation

Postoperative transabdominal ultrasonography and transvaginal observation with an endoscope were performed to monitor the uterus perfused only by uterine vessels on one side. Postoperative resumption of menstruation was also monitored.

### Statistical Analysis

A linear regression model was constructed for examining correlations between the outcome measurement of uterine perfusion time and uterine and ovarian vessels as two explanatory variables. The significance level was 5% and all tests were two-sided. The analyses of this study were performed with SAS statistical software, version 9.2.

## Results

### Intraoperative ICG Fluorescent Angiography

**Figure 3:**
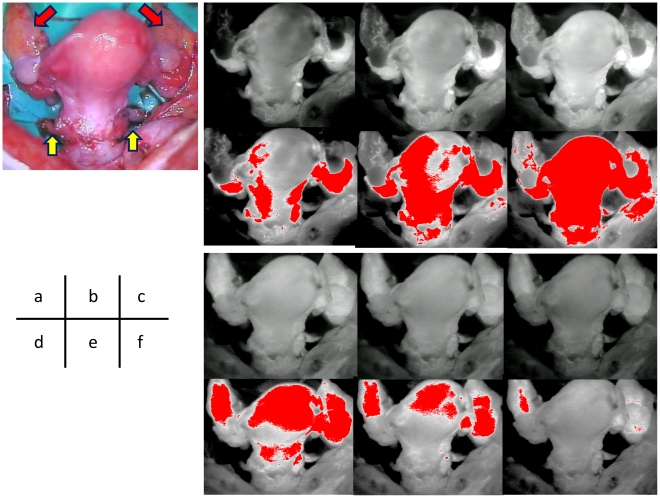
Temporal distribution of uterine blood flow observed by ICG fluorescence imaging after (a) 5 s, (b) 8 s, (c) 12 s, (d) 35 s, (e) 45 s, and (f) 60 s. Upper: ICG fluorescence imaging; lower: Regions with fluorescence intensity ≥100 are shown in red. Uterine artery/vein (yellow arrow) and ovarian artery/vein (red arrow).

ICG fluorescence imaging with a PDE enabled intraoperative real-time observation of uterine hemodynamics. Analysis of the temporal changes in the normal uterus (Fig. 3) showed that ICG expanded from the uterine cervix to the uterine corpus through the uterine artery and was excreted from the uterus through the ovarian vein when full blood flow was observed in the entire uterus.

### Changes in Perfusion Intensity Over Time

**Figure 4:**
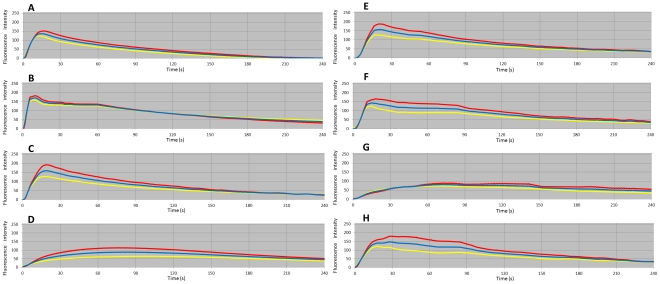
Time-intensity curves for different patterns of vessels for uterine perfusion. A Bilateral uterine + ovarian + vaginal vessels. B Bilateral uterine + ovarian vessels. C Bilateral uterine vessels. D Bilateral ovarian vessels. E Right uterine vessel + right ovarian vessel. F Right uterine vessel + left ovarian vessel. G Right ovarian vessel, H right uterine vessel. Red: uterine corpus, yellow: uterine cervix, blue: average of uterine corpus and cervix.

Temporal changes in perfusion intensity obtained by ICG fluorescence imaging were recorded with ROI software for all patterns of blood vessels. The resulting time-intensity curves (Fig. 4) showed the average intensity in the regions of the uterine corpus and uterine cervix, and in the entire uterus. The time needed to reach perfusion maximum, marked as T_max_, and the time of perfusion decay by half, T_1/2_, were obtained from the curve (Table 2). When all three supplying vessels–uterine vessels, ovarian vessels, and vaginal vessels–were patent, the baseline averaged T_max_ was 14.8 seconds and T_1/2_ was 53.4 seconds. The results from subsequent patterns were also obtained according to different vessels and their numbers of patency. Under the same pattern of selected supplying vessels, the curves for the corpus and cervix illustrated similar tendencies of perfusion.

**Table 2:** T_max_ and T_1/2_ values in time-intensity curves for perfusion of the entire uterine region with different patterns of blood vessels.

Pattern	T_max_ (s)	T_1/2_ (s)
A	14.8	53.4
B	11.6	104.6
C	19.7	73.4
D	75	166.8
E	19.8	89.4
F	18.1	118.6
G	72.6	186.4
H	28	105.8

It was noted, from Table 2, that T_max_ was shorter for patterns with increased numbers of uterine vessels patency. Furthermore, in patterns D and G, which only included ovarian vessels, values of T_max_ and T_1/2_ were strikingly higher when compared with other patterns, suggesting poor uterine perfusion. T_max_ did not differ remarkably between pattern E, in which a uterine artery/vein and ovarian artery/vein on the same side were selected, and pattern F, in which those on opposite sides were selected. Multiple regression analysis of association between uterine perfusion and two nutrient vessels in the cynomolgus macaque showed that uterine vessels were significantly related to T_max_ (P = 0.008), but that ovarian vessels were not significantly related to T_max_ (P = 0.588).

### Postoperative Evaluation of the Uterus

**Figure 5:**
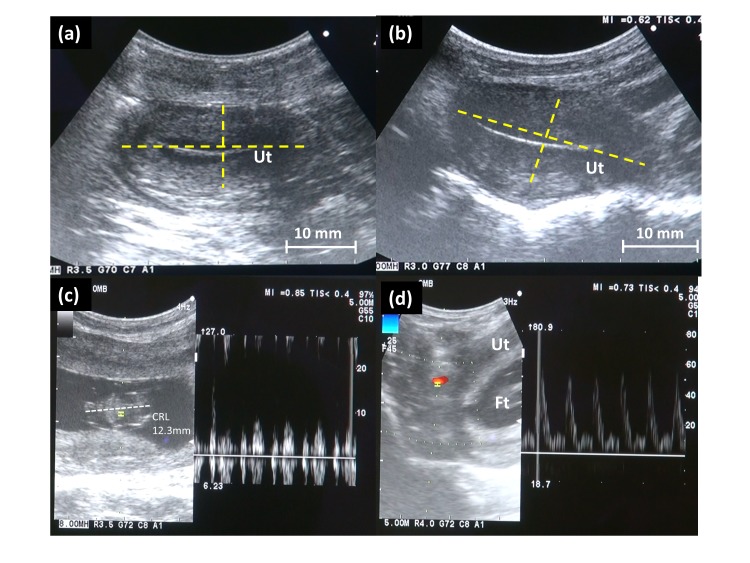
Postoperative evaluation of the uterus. (a) Transabdominal ultrasonography at the time of surgery showed the size of the uterine corpus was 25.2 × 13.1 mm (long axis × anteroposterior diameter) (b) The size of uterine corpus at 2 months after surgery was 25.0 × 13.8 mm, showing no major change in the size of the uterus. Ut: uterus. (c) At 37 days after mating, transabdominal ultrasonography revealed a viable fetus with crown-rump length 12.3mm and regular fetal heart beat. (d) The right uterine artery flow using Doppler ultrasonography was satisfactory and no collateral vessels toward the uterus were shown. Ut: uterus, Ft: fetus.

Findings on transabdominal ultrasonography showed that the size of the uterine corpus was 25.2×13.1 mm (long axis × anteroposterior diameter) at the time of surgery, and this size showed no major change 2 months after surgery (25.0×13.8 mm) (Fig. 5a,b). The RI, PI, and PSV of the right uterine artery using Doppler ultrasonography were 0.615, 0.928, and 28.2 cm/s, respectively, preoperatively; and 0.618, 0.989, and 32.5 cm/s, respectively, at 2 months after surgery, again showing no major change. Transvaginal observation with an endoscopy performed showed a reddish uterine cervix. Periodic menstruation resumed on postoperative day 44.

The cynomolgus macaque underwent natural mating after three normal menstrual cycles. At 37 days after mating, transabdominal ultrasonography revealed a viable fetus with crown-rump length 12.3mm and regular fetal heart beat (Fig. 5c). The right uterine artery flow using Doppler ultrasonography was satisfactory and no collateral vessels toward the uterus were shown (Fig. 5d). At 68 days after mating, the pregnancy is ongoing with favorable fetus well-being.

## Discussion

ICG fluorescence imaging is an angiographic technique that is simple to use and is minimally invasive. As a visualization agent, ICG has the advantages of no uptake by peripheral tissues, rapid elimination from blood, potential for repeated use in tests, and comparatively high safety with few side effects. However, ICG cannot be used in patients with iodine allergy. We showed the value of ICG fluorescence imaging for confirming a site of anastomosis and blood flow in a transplanted uterus of cynomolgus macaque [29]. However, the hemodynamics and nutrient vessels of the uterus have not been previously examined using ICG fluorescence imaging. Therefore, with the aim of examining the hemodynamics in the uterus, we investigated differences in uterine perfusion using different blood vessels that supply nutrients to the uterus using ICG fluorescence imaging during surgery in a cynomolgus macaque.

The uterus has six supply vessels (ovarian, uterine, and vaginal). In abdominal radical trachelectomy, only 2 ovarian vessels have been used to maintain blood supply to the uterus in some cases in which pregnancy and child birth were subsequently successful. Thus, these reports suggest that the ovarian vessels, or 2 out of the 6 uterine vessels, are required to maintain uterine viability [6]. In contrast, it has also been suggested that premature birth or intrauterine growth retardation might occur if the uterine arteries are not preserved, and recent reports support the preservation of uterine artery [7]. For organ perfusion, the artery system that provides the blood supply and the vein system that is responsible for perfusion are both important. The uterine arteries are the main contributors to the blood supply to the uterus. However, in uterine blood perfusion, it is difficult to determine which veins (ovarian vessels, uterine vessels, and deep uterine vein) [41], [42] are most important. In uterine transplantation experiments, Enskog et al. used the ovarian vein and Kisu et al. used the deep uterine vein for uterine perfusion. The temporal distribution of uterine blood flow observed by ICG fluorescence imaging in the current study showed uterine hemodynamics in which the blood flow from the uterine artery perfused mainly to ovarian veins, rather than uterine veins. This suggests that ovarian veins mainly contribute to uterine perfusion during surgery because the diameters of uterine veins are small and some patients may have no uterine veins. Postoperatively, uterine perfusion was achieved only with one uterine vein, and menstruation resumed. Thus, it appears that only one uterine vein is sufficient to maintain the viability of the uterus in some cases. However, further data are required to determine whether one uterine vein is enough to maintain uterus viability in all cases.

T_max_ for intensity was used as a marker of the blood supply to the uterus. The T_1/2_ of intensity could also have been used as a marker of uterine perfusion, but this marker may be inappropriate because it could be affected by a decrease in the plasma elimination rate due to uterine blood supply and repeated administration of ICG, in addition to uterine perfusion. The results based on T_max_ suggested that the uterine blood supply was mainly controlled by uterine vessels, and not by ovarian vessels, and was favorable with a higher number of connected vessels. Menstruation in this cynomolgus macaque was recovered after surgery, showing uterine viability. This suggests that one uterine vessel may be sufficient to maintain uterine perfusion in cynomolgus macaque. However, it may be better to keep two uterine vessels because it may become difficult to maintain the viability of the uterus if a single uterine vessel is stenosed or obstructed. In addition, T_max_ was clearly longer when the blood supply was provided by ovarian vessels only. Therefore, uterine blood supply in trachelectomy and uterine transplantation may require perfusion with at least two uterine arteries.

ICG fluorescence imaging enabled real-time observation of uterine hemodynamics. This method should be useful for confirmation of blood flow in preserved uterine arteries or anastomosed vessels and for evaluation of uterine perfusion in trachelectomy and uterine transplantation. However, this study was performed in the uterus of a cynomolgus macaque and the results may differ from those in a human uterus, although cynomolgus macaque is a similar primate to humans. In addition, only one primate animal was examined in this study due to ethical considerations regarding animal protection, and this makes it difficult to assess the reproducibility of our results. Therefore, we exhaustively searched the literature for studies on irregularity of uterine blood flow, and found that uterine blood flow can be affected by menstrual cycle [2], estrogen/progesterone ratio [43], ovarian artery-to-uterine artery anastomoses in women undergoing fibroid embolization [4], uterine area [2], [3], [44], supplementary flow to the uterus from the vaginal artery [3], [44], and a preserved or non-preserved uterine artery in trachelectomy [6], [7], [10], [45]. However, no data are available on the reproducibility in one subject or for the irregularity of uterine blood flow measured among individuals under the same conditions. Therefore, the results in the current study should be interpreted carefully with regard to reproducibility. Changes in uterine hemodynamics in pregnant subjects also require examination.

The current study was performed as an exploratory investigation of ICG fluorescence imaging for evaluation of uterine blood flow and further data collection is required. However, we conclude that intraoperative real-time observation using ICG fluorescence is useful for evaluation of uterine hemodynamics. Most importantly, our results suggested that uterine vessels, rather than ovarian vessels, are responsible for uterine flood flow, and that even one uterine vessel may be sufficient to maintain uterine viability.

## Supporting Information

Figure S1 **Supplementary video image for time-intensity curves prepared by ROI software to show temporal changes in ICG fluorescence intensity.**
(WMV)
